# Programmable Complex Shape Changing of Polysiloxane Main-Chain Liquid Crystalline Elastomers

**DOI:** 10.3390/molecules28124858

**Published:** 2023-06-20

**Authors:** Yuhe Zhang, Xiuxiu Wang, Wenlong Yang, Huixuan Yan, Xinyu Zhang, Dongxu Han, Yifan He, Chensha Li, Liguo Sun

**Affiliations:** 1 Key Laboratory of Functional Inorganic Material Chemistry, Ministry of Education of the People’s Republic of China, Heilongjiang University, Harbin 150080, China; 2 Department of Applied Science, Harbin University of Science and Technology, Harbin 150080, China; 3 Institute of Regulatory Science, Beijing Technology and Business University, Beijing 100048, China; 4 Key Laboratory of Chemical Engineering Process and Technology for High-Efficiency Conversion School of Chemistry and Material Science, Heilongjiang University, Harbin 150080, China

**Keywords:** polysiloxane liquid crystalline elastomer, programmable shape morphing, two-way network memory, mechanical programming process, two-step crosslinking

## Abstract

Liquid crystal elastomers (LCEs) are shape-morphing materials whose large and reversible shape transformations are caused by the coupling between the mobile anisotropic properties of liquid crystal (LC) units and the rubber elastic of polymer networks. Their shape-changing behaviors under certain stimuli are largely directed by the LC orientation; therefore, various strategies have been developed to spatially modulate the LC alignments. However, most of these methods are limited as they require complex fabrication technologies or have intrinsic limitations in applicability. To address this issue, programmable complex shape changes in some LCE types, such as polysiloxane side-chain LCEs, thiol-acrylate main-chain LCEs, etc., were achieved by using a mechanical alignment programming process coupled with two-step crosslinking. Here, we report a polysiloxane main-chain LCE with programmable 2- and 3D shape-changing abilities that were created by mechanically programming the polydomain LCE with two crosslinking steps. The resulting LCEs exhibited a reversible thermal-induced shape transformation between the initial and programmed shapes due to the two-way memory between the first and second network structures. Our findings expand on the applications of LCE materials in actuators, soft robotics, and smart structures where arbitrary and easily programmed shape morphing is needed.

## 1. Introduction

Liquid crystalline elastomers (LCEs), which belong to a class of outstanding stimuli-responsive materials, are liquid crystal (LC) moieties that are incorporated in polymers with lightly crosslinked networks, in which the LC moieties with predesigned orientations are covalently bonded on the side of polymer backbones (side-chain LCEs) or are directly linked to the polymer backbones (main-chain LCEs) [1,2,3,4,5,6]. The synergy of the self-organization property of LC systems and the polymeric network elasticity allows the LCEs to reversibly exhibit a large and anisotropic dimensional change when external energy stimuli are applied [1,2,3,4,5,6], which thus makes them an appealing material in diverse application fields including soft actuators [7,8,9,10,11,12,13,14,15,16], soft robotics [17,18,19,20,21,22,23,24], artificial muscles [25,26,27,28,29,30,31,32], biomedical devices [33,34,35,36,37,38,39,40,41,42], etc.

The shape changes in LCEs are governed by the coupling between the LC orientations and anisotropic polymer networks, and they rely on a reversible nematic–isotropic transition that is associated with LC moieties. When heated above the nematic–isotropic transition temperature (*T_ni_*), the alignment order of the LC moieties is disrupted, and the LCE transfers from the nematic LC phase into the isotropic state, which drives the macroscopic shape change. Upon cooling below *T_ni_*, the alignment order and LC phase are recovered, and the LCE reverts to its initial shape [1]. The spatial distribution of the LC molecule alignment determines the shape-shifting behavior of the LCEs. Uniaxial alignment can only lead to simple shape-morphing modes such as in-plane contraction and elongation or bending and unbending [1,2,3], and these simple modes cannot meet the development requirements for versatile 3D shape-morphing modes.

More diverse shape morphing in LCE materials can be achieved by imprinting complex LC orientations into LCE networks. A variety of in situ LC molecule alignment techniques, such as surface treatment [43,44,45,46,47], microfluidic processes [48,49,50], photoalignment [6,51,52,53], and the use of magnetic [54,55,56] or electric fields [1], have been employed to achieve spatial control of the LC alignment, which can bring about complex shape morphing. Recently, direct ink-writing 3D printing has been introduced to fabricate LCEs with programmed spatial LC orientations that are induced by shear stress during a filament extrusion and result in 3D shape changes [8,57,58,59,60,61]. These strategies have their respective benefits, but they often have problems regarding their complexity and application limitations; for example, they require tedious orientation technology, monotonous geometries, sophisticated setups, etc., and this is because they generally require the LC orientation profiles to be determined before they can produce the desired shape changes. In recent years, dynamic covalent bonds have been introduced into LCEs to prepare covalent-adaptable LCE networks, which enables LC orientations to be programmed. When an LCE is subjected to mechanical load, an applied stimulus, such as heating or UV irradiation, can trigger an exchange reaction in which the covalent bonds undergo cleavage and rearrangement. After removing the stimulus, the LCE retains the mechanically programmed alignments of the LC moieties in the network [17,62,63,64,65,66,67,68]. The approach of incorporating dynamic covalent bonds into LCEs is robust and facile when fabricating LCE materials with complex shapes and programmable shape-morphing behaviors. However, external stimuli and catalysts or initiators are often needed to drive the bond-exchange reaction; thus, the materials’ performances may be prone to deterioration after they have been repeatedly used.

Finkelmann et al. first developed mechanical alignment to prepare uniaxially aligned monodomain LCEs [69]. They used two-step crosslinking combined with mechanical alignment, and this method has been commonly used to prepare many uniaxially aligned LCE materials. In our previous work, we directly programmed complex 2D shape changes in a classic side-chain polyhydrosiloxane LCE by using this method [70]. The reason that this method works is due to the two-way memory between the first- and second-network structures that are formed during the two-step crosslinking process. The two-way network memory induces two-way shape memory. During the thermal-triggered phase transition between the LC phase and isotropic state, the LCE can reversibly change between the initial shape formed during the first crosslinking step and any another shape programmed by the mechanical processes and fixed by the second crosslinking step [70]. This demonstrated performance is similar to that of shape memory polymers (SMPs), whose stimuli-responsive shape changes can be readily achieved by utilizing mechanical programming processes [71]. A temporary shape can be directly imprinted in an SMP under external stress and elevated temperature, and this shape can remain after quenching. Upon heating, the SMP recovers its initial shape, and the programmed shape transformation is completed. Since our that work [70], researchers have achieved SMP-like programmable complex shape changes in other types of LCEs, such as thiol-acrylate main-chain LCEs, alkylamine-acrylate main-chain LCEs, etc., by using methods based on two-step crosslinking combined with a mechanical alignment [21,72,73,74]. Polysiloxane-typed LCEs are one of the widely applied LCE types. Thus, further developing programmable complex shape changes in polysiloxane LCEs is important.

We directly programmed complex reversible shape morphing in polysiloxane main-chain LCEs by using an approach based on two-step crosslinking combined with mechanical alignment. Main-chain LCEs can exhibit much higher mechanical and strain actuation properties than side-chain LCEs due to the direct coupling of the LC moieties to polymer backbone chains [1]; thus, they should demonstrate more sophisticated 3D-programmable shape morphing behaviors compared with our first side-chain LCEs [70]. During the first crosslinking step, a partially crosslinked polydomain LCE was generated through the hydrosilylation polyaddition and crosslinking reaction driven by ultrasonic sonochemistry processes [75]. Then, via stretching, pressing, stamping, or embossing, we mechanically programmed the LCE to be another 1-, 2-, or 3D-shaped LCE. Lastly, the LC alignment distribution and the programmed shape of the material were locked by the second thermal crosslinking step. Reversible transformations of the LCEs between the initial shapes and the programmed shapes were successfully demonstrated when exposed to heat, which resulted in direct mechanically programmed shape morphing. This facile and versatile strategy of realizing programmable shape morphing by utilizing a two-way network memory necessitates no foresight of the required LC orientation profile and is not limited to the fabricated shapes of the LCE materials, and thus this strategy has the potential to facilitate the design and application of LCE materials in sophisticated smart devices and multifunctional structures where arbitrary and easily programmed shape morphing is needed.

## 2. Results and Discussion

**Scheme 1:**
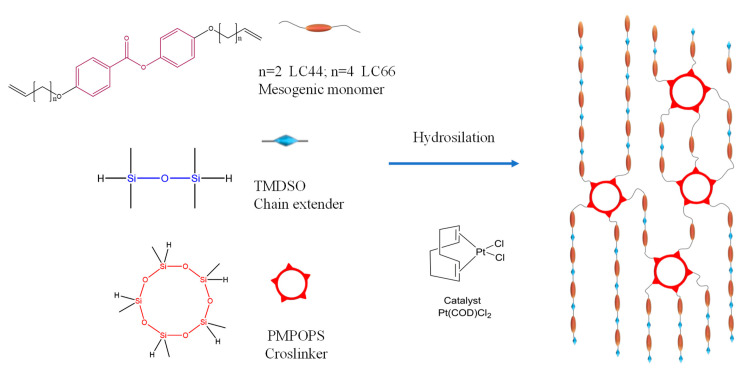
Illustration of precursor reactant molecules and the synthesis of polysiloxane main-chain LCE network via hydrosilation reaction.

Regarding the strategy we used to directly program complex reversible shape changes in polysiloxane main-chain LCEs, the fundamental logic was to build two-step network structures in the LCE matrix and utilize the two-way network memory, which did not require us to determine the LC orientation profiles to produce the desired shape changes. The polysiloxane main-chain LCE network was synthesized via sol-gel processed hydrosilation [1,75], as illustrated in Scheme 1. The precursor reactants included the monomers of the mesogens LC44 and LC66, chain extender EDDET, and crosslinker PMPOPS. The terminal vinyl bonds of the mesogenic molecules reacted with the Si-H bonds of the chain extender and crosslinker molecules due to a platinic acid catalyst that was employed to provoke the hydrosilation polyaddition polymerization and crosslinking reaction to generate the LCE network. After performing the first crosslinking step, a partly crosslinked polydomain network, in which the mesogens were randomly oriented on average [1], was generated. Then, an external force was applied to reshape the material and establish the spatial alignment distribution of the mesogens. After the second crosslinking step, the network topology with the mesogen alignments and the mechanically programmed material shape was permanently fixed. The procedures used to prepare our LCEs with 1-, 2-, or 3D-programmed shapes were based on the above principle, as described in the experiment section and S-Ⅱ, -III, and -IV of the ESI †.

**Figure 1:**
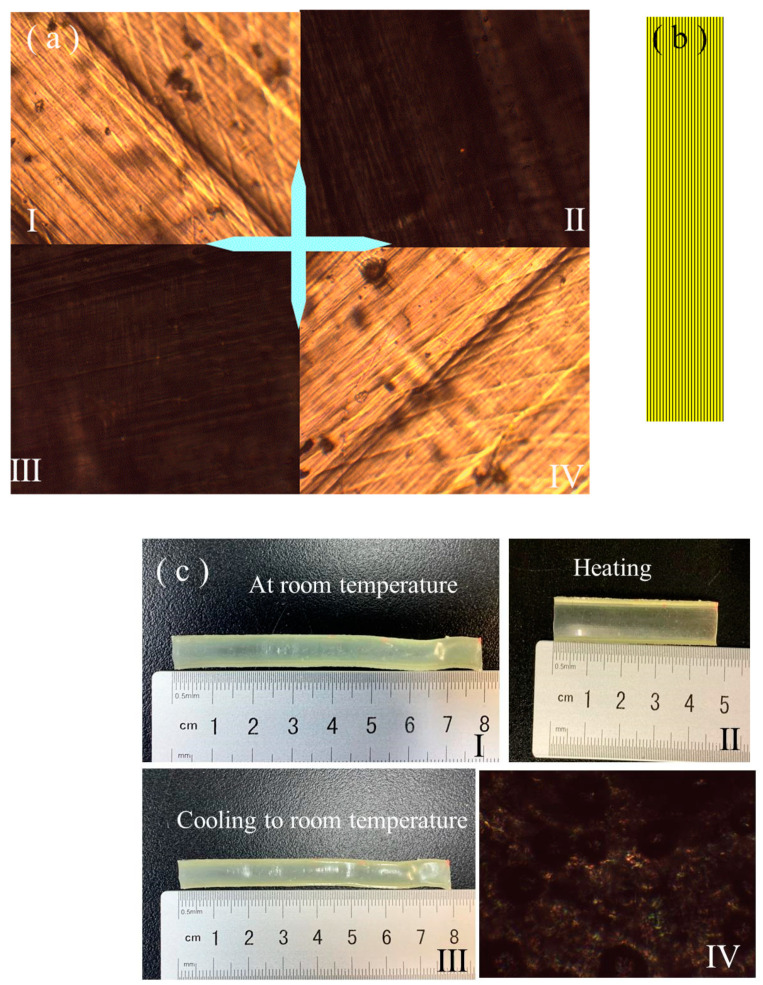
(**a**) POM images of the strip-shaped LCE. The crossed arrows denote the horizontal analyzer and vertical polarizer. (**a-I**) The stretching direction inclined at −45° to the polarizer. (**a-II**) The stretching direction parallel to the polarizer. (**a-III**) The stretching direction parallel to the analyzer. (**a-IV**) The stretching direction inclined at 45° to the polarizer. (**b**) The schematic alignment distribution in the strip-shaped LCE. (**c**) Images of the states of strip-shaped LCE initially on a hot stage at room temperature (**c-I**), when being heated to 80 °C (**c-II**), when being cooled to room temperature (**c-III**), and a POM image of strip-shaped LCE being heated at 80 °C (**c-IV**).

The two fabricated LCEs with 1D-programmed shapes had a rectangular strip and round rod shape, as expounded in S-Ⅱ of the ESI †. Figure 1a shows a POM observation of the strip-shaped LCE. The LCE exhibited the brightest transmittance when its stretching direction was at an angle of ±45° with the polarizer or analyzer (Figure 1(a-I,a-IV)), whereas extinctive transmittance was found when its stretching direction was parallel or perpendicular to the polarizer or analyzer (Figure 1(a-II,a-III)); this revealed that the mesogens were axially aligned along its stretching direction [1], as illustrated in Figure 1b. The thermal actuation behavior of the strip-shaped LCE was examined on a program-controlled hot stage, as shown in the images in Figure 1c. Upon heating to 80 °C, it contracted along the length direction, which was the alignment direction, and expanded along the width direction. Upon cooling to room temperature, it returned to its initial length and width. This reversible thermal-induced deformation was caused by the reversible phase transition between the alignment structure and isotropic state upon undergoing the heating and cooling process [1,2,3,4,5,6]. Upon being heated to 80 °C, the strip-shaped LCE demonstrated a dark pattern under the POM regardless of the angle that it was at relative to the polarizer or analyzer, and this was due to its isotropic state, as shown in Figure 1(c-IV). When the LCE network transitioned from the LC phase to an isotropic state, it contracted along the alignment direction and expanded along the directions that were perpendicular to the alignment [1]. We found that the resulting length and width of the rectangular strip-shaped LCE, after being heated above the *T_ni_* (about 62 °C [75]), were basically consistent with those of the partly crosslinked polydomain LCE with a rectangular strip shape after undergoing the first crosslinking step, as expounded in S-II and illustrated in Scheme S1 of the ESI †. This revealed that the LCE demonstrated two-way shape memory between the initial shape formed after the first crosslinking step and the mechanically programmed shape that was fixed by the second crosslinking step. This two-way shape memory was induced by the two-way network memory between the first- and second-network structure that was generated during the two-step crosslinking process [70].

**Figure 2:**
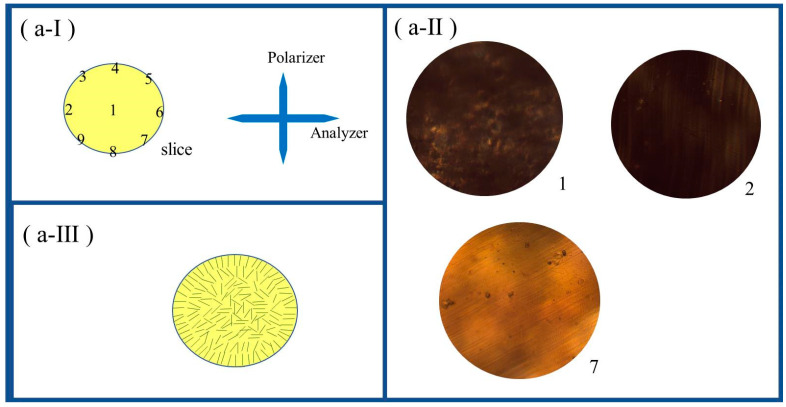

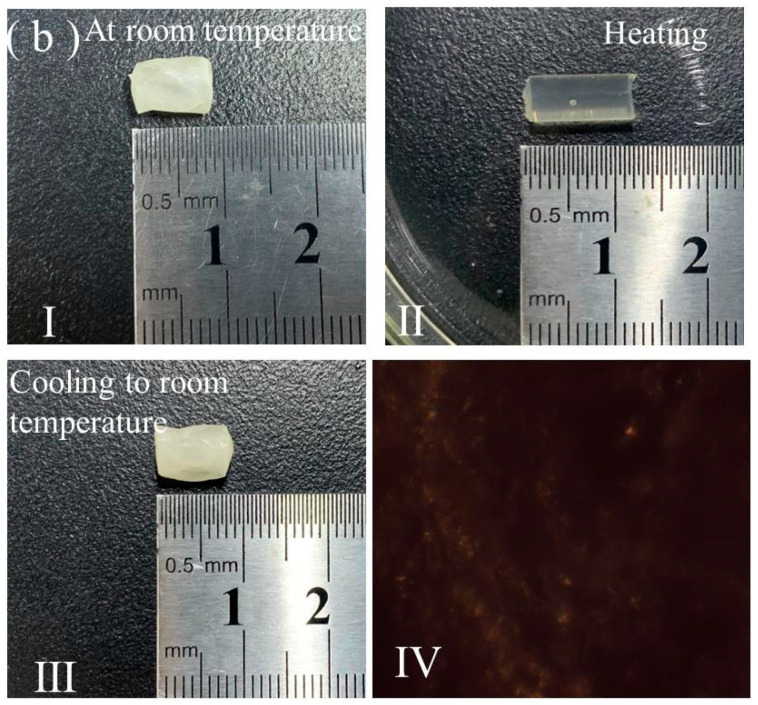
(**a-I**) Schematic illustration of the selected coordinate locations, marked as 1 to 9, in a transversely cut slice of rod-shaped LCE, for POM measurement. The crossed arrows denote the horizontal analyzer and vertical polarizer. (**a-II**) POM images captured at coordinate location 1, 2, and 7. (**a-III**) Schematic illustration of alignment distribution in cross-section of the rod-shaped LCE. (**b**) Images of the states of rod-shaped LCE initially on a hot stage at room temperature (**b-I**), when being heated to 80 °C (**b-II**), when being cooled to room temperature (**b-III**), and a POM image of a transversely cut slice of rod-shaped LCE being heated at 80 °C (**b-IV**).

A transversely cut slice of the rod-shaped LCE was used for the POM measurement. As illustrated in Figure 2(a-I), the coordinate locations 1 to 9 were selected to be observed with the POM. The coordinate location 1 was the center area, whereas the coordinate locations 2 to 9 were near the circumference edge. The coordinate locations 2 and 6 together were located on a line that was parallel to the analyzer and passed through the center dot. The coordinate locations 4 and 8 were located together on a line that was parallel to the polarizer and passed through the center dot. The line connecting with coordinate locations 3 and 7 and the line connecting with coordinate locations 5 and 9 were all at a ±45° angle relative to the analyzer or polarizer and passed through the center dot. The POM measurement result demonstrated that the coordinate locations 2, 4, 6, and 8 exhibited dark transmittance, whereas the coordinate locations 3, 5, 7, and 9 exhibited bright transmittance. Figure 2(a-II) shows the representative dark transmittance of location 2 and the representative bright transmittance of location 7. Location 1 was composed of discrete tiny bright and dark areas, as shown in Figure 2(a-II). The transmittance effects of these coordinate locations were constant after the cut slice was rotated at any angle. The POM measurement showed that for the rod-shaped LCE, the mesogens were aligned along the radial directions in the area near the circumference edge, but the center area had a disordered polydomain structure, as illustrated in Figure 2(a-III). Regarding the polydomain structure, every tiny domain had an alignment structure and birefringence characteristics, but the domains were randomly oriented on average. Thus, the structure demonstrated discrete tiny bright and dark areas under the POM. The images in Figure 2b showed that the rod-shaped LCE exhibited thermal actuation behavior during the program-controlled hot stage. Upon heating to 80 °C, it elongated along the axial direction and became a little thinner along the radial direction. Upon cooling to room temperature, it returned to the initial axial and radial dimensions. This reversible shape-changing mode that occurred during the heating and cooling process also resulted from the alignment distribution. Upon being heated to 80 °C, the cut slice of the rod-shaped LCE exhibited a dark pattern under the POM regardless of the any angle that it was rotated at, and this was due to its isotropic state, as shown in Figure 2(b-IV). We found that the resulting dimensions in the axial height and radial diameter of the rod-shaped LCE, when being heated to 80 °C, were basically consistent with those of the partly crosslinked polydomain LCE with a rod shape after the first crosslinking step, as expounded in S-II and illustrated in Scheme S2 of the ESI †. This revealed that the rod-shaped LCE also demonstrated two-way shape memory between the initial shape formed after the first crosslinking step and the mechanically programmed shape that was fixed by the second crosslinking step.

To determine the two-way shape memory ability of our polysiloxane main-chain LCE materials, several actuation fixity parameters for the 1D-programmed LCEs were defined by leveraging the definition of the actuation fixity parameters of the SMP [71]. The defined actuation fixity parameters are the curing fixity, *f_cure_*; heating deformation fixity, *f_heat_*; and cooling deformation fixity, *f_cool_*, as expounded in S-Ⅴ of the ESI †. The results of our experiment showed that the ultrasonication time, which determined the energy supplied during the first crosslinking step, influenced the actuation fixity parameters. An insufficient ultrasonication time leads to a weaker first crosslinking, and the *f_heat_* will be lowered. An ultrasonication time that is too long leads to too high degree of first step crosslinking, the *f_cure_* and *f_cool_* will be lowered. The used ultrasonication time was the optimum time, as described in S-II of the ESI †. The measured actuation fixities of the strip- and rod-shaped LCEs are listed in Table 1. Table 1 shows that the actuation fixities were superior. Moreover, the measured *f_heat_* and *f_cool_* values of the 1D-programmed LCEs were basically constant after several tens of repeated heating and cooling actuations. Thus, our polysiloxane main-chain LCE materials should be capable of effectively undergoing programmable complex shape morphing.

**Figure 3:**
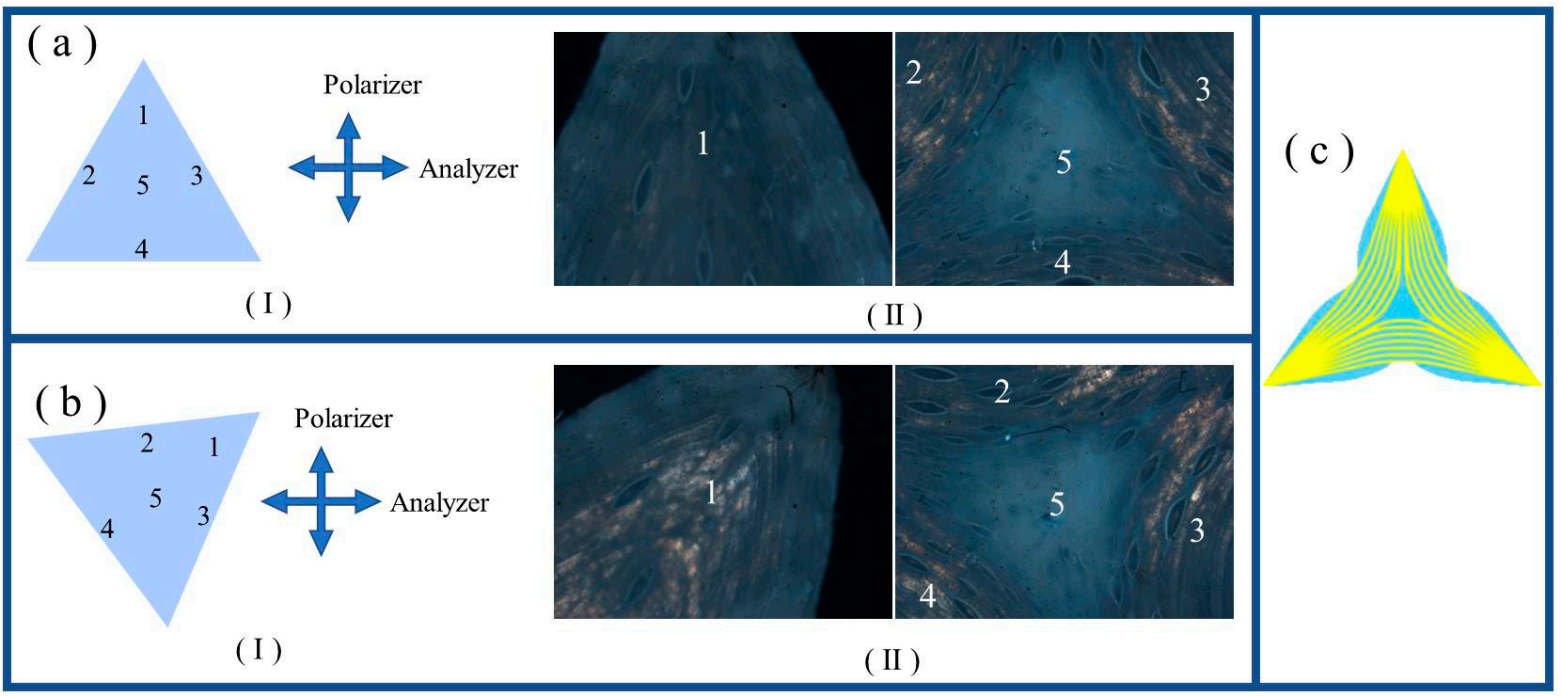
(**a-I**) Schematic illustration of the selected locations, with number markings, in the LCE sample with a regular triangle shape for POM measurement. The crossed arrows denote the horizontal analyzer and vertical polarizer. (**a-II**) POM images captured at the selected locations of regular-triangle-shaped LCE. (**b-I**) Schematic illustration of LCE sample of regular-triangle shape, with locations marked, being rotated right by 45°. (**b-II**) POM images captured at the selected locations of regular-triangle-shaped LCE that was rotated right by 45°. (**c**) Schematic illustrations of alignment distributions in regular-triangle-shaped LCE.

**Figure 4:**
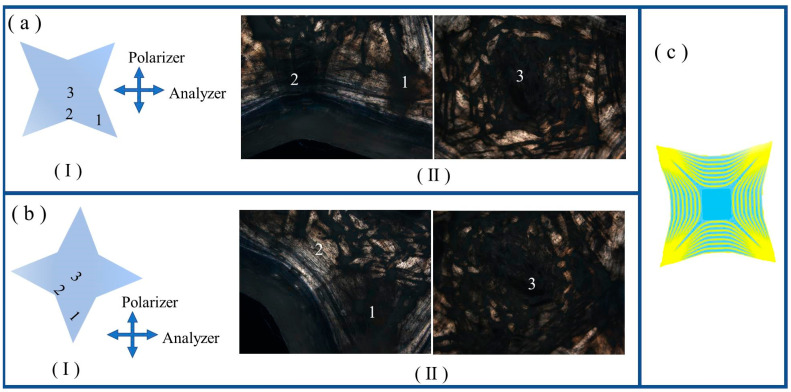
(**a-I**) Schematic illustration of the selected locations, with number markings, in the LCE sample with a four-pointed-star shape for POM measurement. The crossed arrows denote the horizontal analyzer and vertical polarizer. (**a-II**) POM images captured at the selected locations of four-pointed-star-shaped LCE. (**b-I**) Schematic illustration of LCE sample of four-pointed star shape, with location markings, being rotated right by 45°. (**b-II**) POM images captured at the selected locations of four-pointed-star-shaped LCE that was rotated right by 45°. (**c**) Schematic illustrations of alignment distributions in four-pointed-star-shaped LCE.

**Figure 5:**
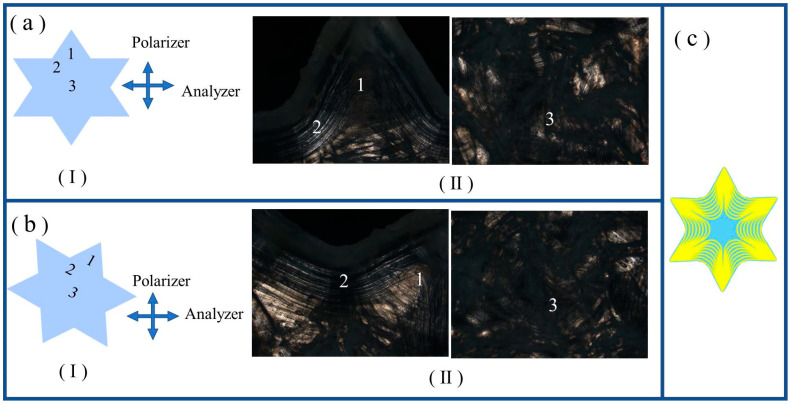
(**a-I**) Schematic illustration of the selected locations, with number markings, in the LCE sample with a six-pointed star shape for POM measurement. The crossed arrows denote the horizontal analyzer and vertical polarizer. (**a-II**) POM images captured at the selected locations of six-pointed-star-shaped LCE. (**b-I**) Schematic illustration of LCE sample of six-pointed star shape, with location markings, being rotated right by 30°. (**b-II**) POM images captured at the selected locations of six-pointed-star-shaped LCE that was rotated right by 30°. (**c**) Schematic illustrations of alignment distributions in six-pointed-star-shaped LCE.

The three fabricated LCEs with 2D-programmed shapes had a regular-triangle, four- and six-pointed-star shape, as expounded in S-III of the ESI †. The partly crosslinked polydomain LCE with a disc shape, which was synthesized during the first crosslinking step, was mechanically programmed to be another shape, either a regular triangle or four- or six-pointed star shape, and then the programmed shape was fixed by the second crosslinking step, as shown in Scheme S3. As illustrated in Figure 3(a-I), for a placed regular triangle-shaped LCE with one sideline parallel to the analyzer, locations 1 to 5 were selected as the representative parts for the POM observation. Location 1 was the top area, which was parallel to the polarizer. Locations 2, 3, and 4 were the middle part between every two adjacent angles. Location 5 was the center area of the regular triangle. The POM images, shown in Figure 3(a-II), demonstrated that locations 1, 4, and 5 exhibited dark transmittance, whereas locations 2 and 3 exhibited bright transmittance. When the regular triangle-shaped LCE was rotated right by 45°, as illustrated in Figure 3(b-I), locations 1, 3, and 4 exhibited bright transmittance, whereas locations 2 and 5 exhibited dark transmittance, as shown in Figure 3(b-II). The alignment distribution in the regular triangle-shaped LCE evidenced by the POM measurement is shown in Figure 3c. At every angle, the alignments were symmetrically distributed with respect to the angular bisector. The alignments in the areas close to the angular bisectors were approximately parallel to the angular bisectors. The alignments in the middle part between any two adjacent angles were basically parallel to the sideline connecting the two adjacent angles. The center area of the regular triangle-shaped LCE had a disordered polydomain structure. As illustrated in Figure 4(a-I), for a placed four-pointed-star-shaped LCE with the two diagonals at a ±45° angle relative to the analyzer or polarizer, locations 1, 2, and 3 were selected as the representative parts for the POM observation. Location 1 was the right lower-star blade. Location 2 was the middle area between the left lower-star blade and right lower-star blade. Location 1 was the center area of the four-pointed star. POM images are presented in Figure 4(a-II); location 1 exhibited bright transmittance, location 2 exhibited dark transmittance, and location 3 was composed of discrete tiny bright and dark areas. When the four-pointed-star-shaped LCE was rotated right by 45°, as illustrated in Figure 4(b-I), location 1 exhibited dark transmittance, location 2 exhibited bright transmittance, and location 3 was still composed of discrete tiny bright and dark areas. The alignment distribution of the four-pointed-star-shaped LCE evidenced by the POM measurement is shown in Figure 4c. In every star blade, the alignments were symmetrically distributed with respect to the bisector. The alignments in the areas close to the bisectors were approximately parallel to them. The alignments in the middle area between every two adjacent star blades were approximately parallel to the line connecting the two blade tips. The center area of the four-pointed-star-shaped LCE had a disordered polydomain structure. As illustrated in Figure 5(a-I), for a placed six-pointed-star-shaped LCE with one diagonal parallel to the polarizer, locations 1, 2, and 3 were selected as the representative parts for the POM observation. Location 1 was the top star blade. Location 2 was the middle area between the top star blade and the left-upper star blade. Location 3 was the center area of the six-pointed star. The POM images presented in Figure 5(a-II) demonstrated that location 1 exhibited dark transmittance, location 2 exhibited bright transmittance, and location 3 was composed of discrete tiny bright and dark areas. When the six-pointed-star-shaped LCE was right rotated by 30°, as illustrated in Figure 5(b-I), location 1 exhibited bright transmittance, location 2 exhibited dark transmittance, and location 3 was still composed of discrete tiny bright and dark areas. The alignment distribution in the six-pointed-star-shaped LCE evidenced by the POM measurement is shown in Figure 5c. In every star blade, the alignments were symmetrically distributed with respect to the bisector. The alignments in the areas close to the bisectors were approximately parallel to the bisectors. The alignments in the middle area between every two adjacent star blades were approximately parallel to the line connecting the two blade tips. The center area of the six-pointed-star-shaped LCE had a disordered polydomain structure.

**Figure 6:**
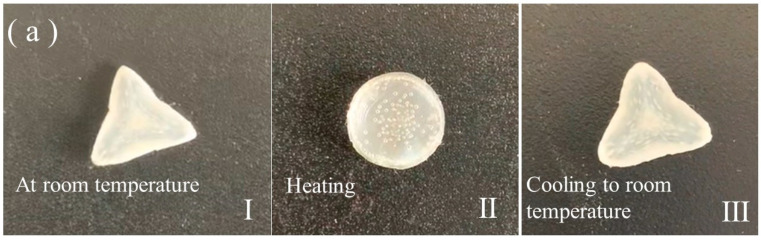

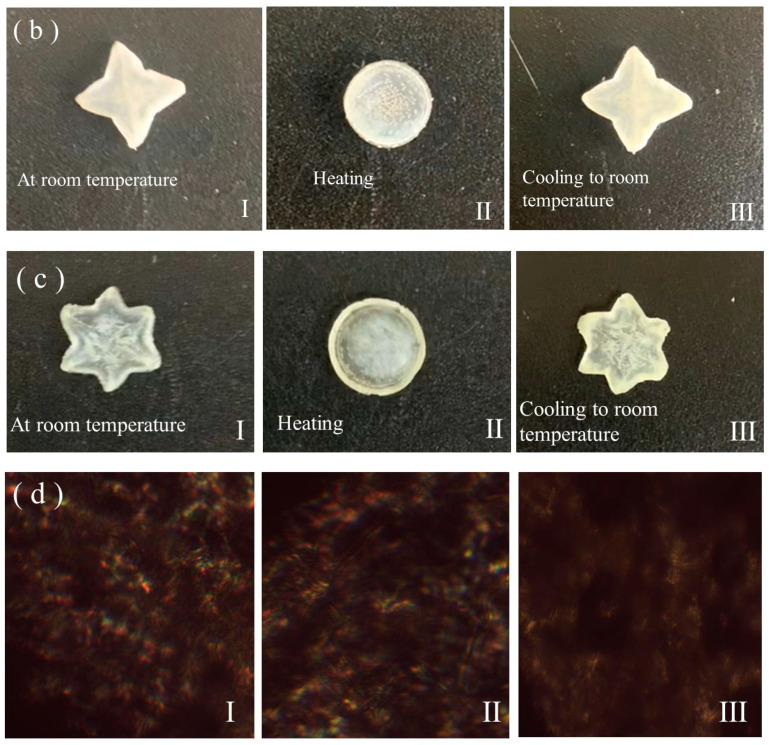
(**a**–**c**) Images of the states of regular-triangle-shaped LCE (**a**), four-pointed-star-shaped LCE, (**b**) and six-pointed-star-shaped LCE (**c**) initially on a hot stage at room temperature (**a-I**–**c-I**), when being heated to 80 °C (**a-II**–**c-II**), and when being cooled to room temperature (**a-III**–**c-III**). (**d**) POM images of the regular-triangle- (**d-I**), four- (**d-II**), and six-pointed-star-shaped LCE (**d-III**) when being heated at 80 °C.

The thermal actuation behaviors of the regular triangle-shaped and four- and six-pointed-star-shaped LCEs were examined on a program-controlled hot stage. As shown in Figure 6a–c, and the S1, S2, and S3 videos of the ESI †, upon heating to 80 °C, the regular triangle-shaped and four- and six-pointed-star-shaped LCEs all changed to be disc shape, whereby the shape and diameter were consistent with the partly crosslinked polydomain LCEs that were synthesized during the first crosslinking step. Upon cooling to room temperature, they regained their original shapes and dimensions. These LCEs with 2D-programmed shapes all demonstrated a strong two-way shape memory performance, although their geometries and mesogen alignment distributions were different. Figure 6d shows the POM observations of the LCEs with a regular triangle and four- and six-pointed star shape upon being heated to 80 °C. They all demonstrated a dark pattern regardless of the angle they were rotated at due to their isotropic state.

**Figure 7:**
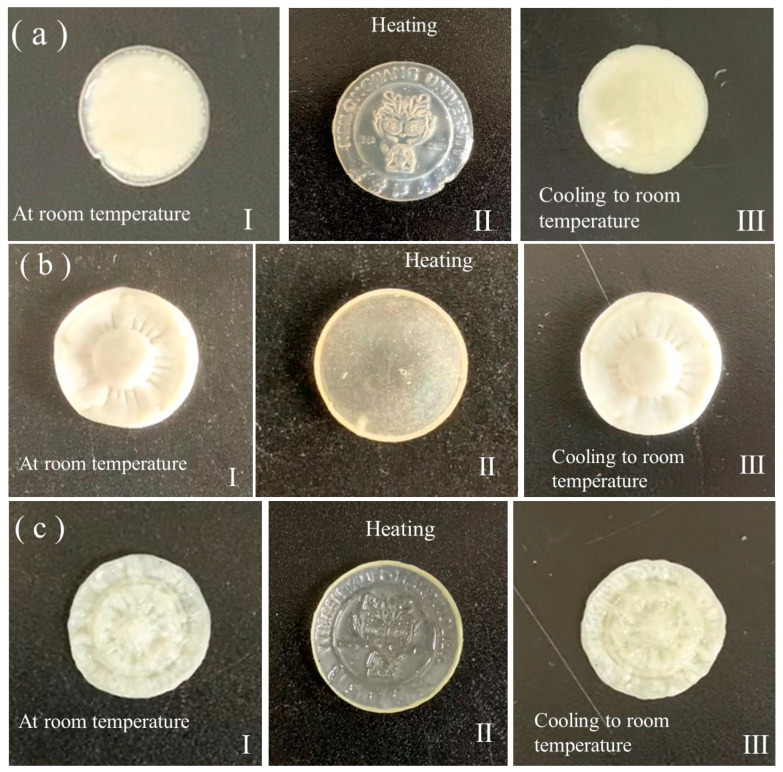
Images of the states of 3D-S-LCE (**a**), 3D-E-LCE (**b**), and 3D-SE-LCE (**c**) initially on a hot stage at room temperature (**a-I**–**c-I**), when being heated to 80 °C (**a-II**–**c-II**), and when being cooled to room temperature (**a-III**–**c-III**).

The fabrications of three LCEs with 3D-programmed shapes, which were nominated as 3D-S-LCE, 3D-E-LCE, and 3D-SE-LCE, are expounded in S-IV of the ESI †. For the fabrication of 3D-S-LCE, the partly crosslinked polydomain LCE that was synthesized in the first crosslinking step had a disc shape with a 3D pattern of the Heilongjiang University logo on one surface, which was stamped with a badge during the first crosslinking step. The patterned surface was mechanically pressed to be flat. Then, the flattened surface was fixed by the second crosslinking step, as shown in Scheme S4. When fabricating 3D-E-LCE, the partly crosslinked polydomain LCE that was synthesized during the first crosslinking step had a disc shape with flat surfaces. A 3D plum blossom was mechanically embossed on its one surface by using a plum blossom plastic mold (no. 1 plastic mold), and then it was fixed by the second crosslinking step, as shown in Scheme S5. When fabricating 3D-SE-LCE, the partly crosslinked polydomain LCE synthesized during the first crosslinking step had a stamped 3D pattern of the Heilongjiang University logo on one surface. The patterned surface was mechanically embossed to be another 3D plum blossom shape, and this was performed by using another plum blossom plastic mold (no. 2 plastic mold), and then it was fixed by the second step crosslinking, as shown in Scheme S6. The thermal actuation behaviors of 3D-S-LCE, 3D-E-LCE, and 3D-SE-LCE were examined on a program-controlled hot stage, as shown in Figure 7 and Figures S4–S6 of videos in the ESI †. For the 3D-S-LCE, the initial stamped 3D pattern of the Heilongjiang University logo that was formed during the first crosslinking step reappeared on its surface when it was heated to 80 °C. Upon cooling to room temperature, the surface resumed its flat state. For the 3D-E-LCE, upon heating to 80 °C, the 3D plum blossom disappeared, and the surface became flat. Upon cooling to room temperature, the surface resumed its 3D plum blossom pattern. For the 3D-SE-LCE, the 3D plum blossom pattern on its surface changed to the 3D pattern of the Heilongjiang University logo that was formed during the first crosslinking step. Upon cooling to room temperature, the surface changed back to the 3D plum blossom pattern. The fabricated LCEs with 3D-programmed shapes had complicated topological structures and mesogen alignments with spatial distributions that were difficult to clearly measure. Therefore, foreseeing their shape-changing modes by determining the LC orientation profiles is very difficult. However, by using the two-way shape memory, reversible shape morphing between arbitrary complicated 3D shapes that are predetermined by mechanical shaping procedures can be conveniently programmed and achieved.

## 3. Materials and Methods

### 3.1. Material Preparations

The mesogens (LC units), which were 4-(but-3-en-1-yloxy)phenyl 4-(but-3-en-1-yloxy)benzoate (LC44) and 4-(hex-5-en-1-yloxy)phenyl 4-(hex-5-en-1-yloxy)benzoate (LC66), were synthesized by employing the methods outlined in our previously reported work [75]. The NMR measurement results of LC44 and LC66 were expounded in S-I and are shown in Figures S1 and S2 of the ESI †. The chain extender, cyclic siloxane crosslinker, and commercial platinum catalyst, which were 1, 1, 3, 3-teramethydisiloxane (TMDSO), 2, 4, 6, 8, 10-pentamethyl-1, 3, 5, 7, 9, 2, 4, 6, 8, 10-pentaoxapentasilecane (PMPOPS), and dichloro (1,5-cyclooctadiene) platinum (II) (Pt(COD)Cl_2_), respectively, were purchased from Aldrich (St. Louis, MO, USA). The catalyst solution was prepared by using the methods outlined in our previous work [75].

The precursor reactant solutions were prepared by dissolving the mesogenic monomers LC44 and LC66, chain extender TMDSO, and crosslinker PMPOPS in a toluene solvent. Table 2 lists the reactant amounts, toluene solvent volume, and catalyst solution that were used to prepare the polysiloxane main-chain LCEs with 1-, 2-, or 3D-programmed shapes. The processes of preparing the LCEs are described in S-Ⅱ, -III, and -IV of the ESI † and are illustrated in Scheme S1 to S6 of the ESI †.

### 3.2. Characterization Methods

^1^H NMR spectra were recorded by using a Bruker HW400 MHz spectrometer (AVANCE III), CDCl_3_ as the solvent, and CHCl_3_ (δ 7.26) and DMSO (δ 2.50) as the interior reference. A polarizing optical microscope (POM, SMZ 1500, Nikon Instruments Co. Tokyo, Japen) was used to determine the mesogenic alignment distributions in the LCE matrices, whereby we measured the transmittance of a probe light through two crossed polarizers and analyzers with the LCE sample set between them.

## 4. Conclusions

Herein, we presented the result of directly programming complex reversible shape morphing in polysiloxane main-chain LCEs by using mechanical programming processes, which was inspired by the principle of using mechanical programming processes to achieve programmable shape-changing behavior in SMPs. Polydomain LCEs were first synthesized with distinct geometries that were not limited to flat shapes via a sol-gel processed hydrosilation reaction. The programmed arbitrary-shaped LCEs were prepared via mechanical programming, such as by stretching, pressing, stamping, or embossing the polydomain LCEs, and the subsequent second crosslinking step. After the second crosslinking, which permanently fixed the programmed LCE shape and the alignment distribution of the mesogens formed during the mechanical programming process, the resulting LCEs exhibited reversible shape morphing between the initial and programmed shapes, which were induced by the LC phase transition upon heating and cooling. This facile and versatile strategy of realizing programmable shape morphing utilizes the two-way memory between the first and second network structure generated during the two-step crosslinking process; thus, the spatial distribution of the mesogen alignments does not need to be meticulously modulated. This strategy is suitable for polysiloxane main-chain LCEs due to their high processability, elasticity, and reactivity during preparation, which allows them to be easily fabricated into different initial shapes and programmed into various complex shapes. Our work widens the prospects for the design and application of LCE materials in smart actuators, biomedical devices, soft robotics, multifunctional structures, etc., where arbitrary and easily programmed shape morphing is needed.

## Figures and Tables

**Table 1:** The actuation fixities of 1D-programmed LCEs.

Programmed Shape	*f_cure_*	*f_heat_*	*f_cool_*
Rectangular strip	98.1%	98.8%	≈100%
Round rod	97.9%	98.3%	≈100%

**Table 2:** The used amounts of precursor reactants for LCE preparations.

Programmed Shape	Reactant Amounts					
	LC44	LC66	TMDSO	PMPOPS	Toluene Solvent (µL)	Catalyst Solution (µL)
1D shape: strip shape	0.1692 g (0.5 mmol)	0.1973 g (0.5 mmol)	0.1072 g (0.8 mmol)	0.0268 g (0.08 mmol)	1300	25
1D shape: rod shape	0.0423 g (0.125 mmol)	0.04933 g(0.125 mmol)	0.0268 g (0.2 mmol)	0.0067 g (0.02 mmol)	330	8
2D shape: six or four-pointed star or regular triangle	0.0846 g (0.25 mmol)	0.09865 g (0.25 mmol)	0.0536 g (0.4 mmol)	0.0134 g (0.04 mmol)	650	12
3D shape: 3D-S-LCE, 3D-E-LCE, or 3D-SE-LCE”	0.0846 g (0.25 mmol)	0.09865 g (0.25 mmol)	0.0536 g (0.4 mmol)	0.0134 g (0.04 mmol)	650	12

## Data Availability

Not applicable.

## Supplementary Materials

The following supporting information can be downloaded at: https://www.mdpi.com/article/10.3390/molecules28124858/s1. † Electronic supplementary information (ESI) available: Supplementary Video S1: video of thermal-induced programmable shape change in regular-triangle-shaped LCE, speed: 3×; Supplementary Video S2: video of thermal-induced programmable shape change in four-pointed-star-shaped LCE, speed: 3×; Supplementary Video S3: video of thermal-induced programmable shape change in six-pointed-star-shaped LCE, speed: 3×; Supplementary Video S4: video of thermal-induced programmable shape change in 3D-S-LCE, speed: 3×; Supplementary Video S5: video of thermal-induced programmable shape change in 3D-E-LCE, speed: 3×; Supplementary Video S6: video of thermal-induced programmable shape change in 3D-SE-LCE, speed: 3×. Figure S1: 1H NMR spectrum of mesogenic monomer LC44; Figure S2: 1H NMR spectrum of mesogenic monomer LC66; Scheme S1: Illustration of the preparation protocol of strip-shaped LCE; Scheme S2: Illustration of the preparation protocol of rod-shaped LCE; Scheme S3: Illustration of the preparation protocol of LCE with a shape of six-pointed star, four-pointed star or regular triangle; Scheme S4: Illustration of the preparation protocol of 3D-S-LCE; Scheme S5: Illustration of the preparation protocol of 3D-E-LCE; Scheme S6: Illustration of the preparation protocol of 3D-SE-LCE; Scheme S7: Illustration for the calculation of actuation fixity of the strip-shaped LCE; Scheme S8: Illustration for the calculation of actuation fixity of the rod-shaped LCE.
