# Supplementary material for: Programmable Complex Shape Changing of Polysiloxane Main-Chain Liquid Crystalline Elastomers
Source: Molecules. 2023 Jun 20;28(12):4858. doi: 10.3390/molecules28124858 (PMC10303392; doi:10.3390/molecules28124858)
Supplement: Supplementary file 1 [file molecules-28-04858-s001.zip › Support information - modification.pdf]

# Supporting Information

# Programmable Complex Shape Changing of Polysiloxane Main-Chain Liquid-Crystalline Elastomers

## S-I: The NMR measurement of mesogenic monomer LC44, LC66.

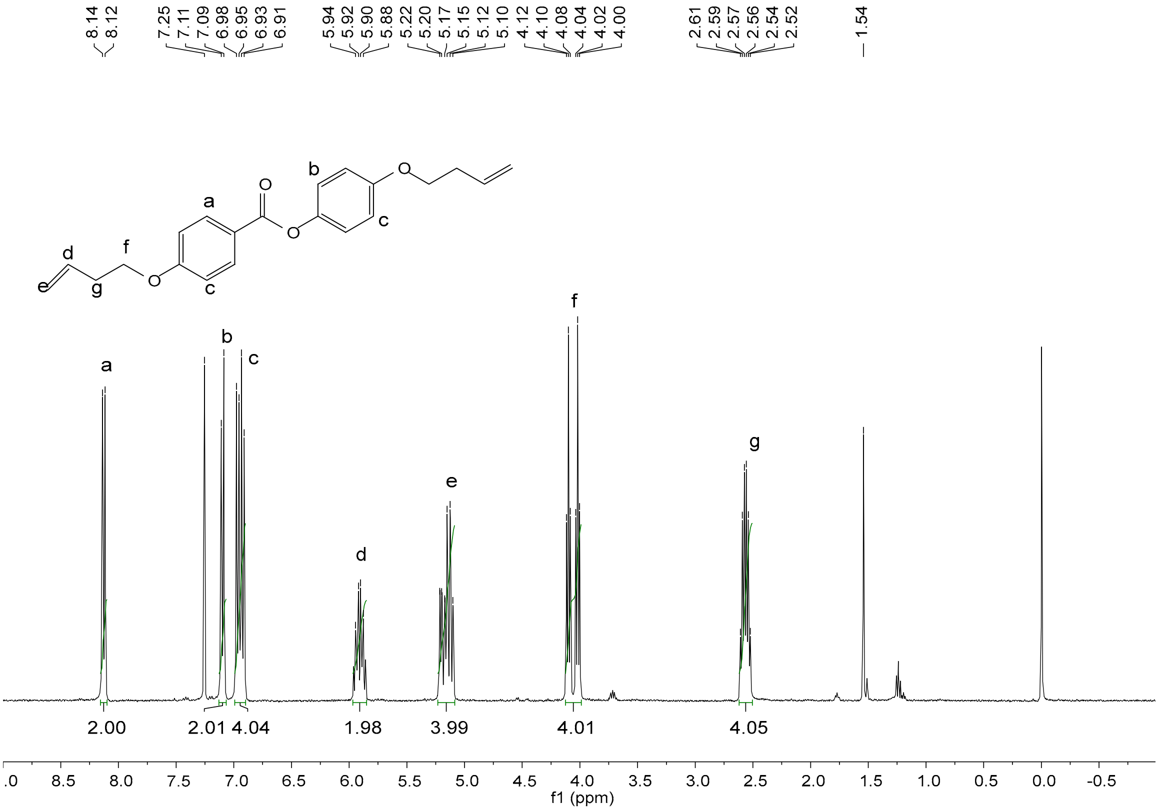


**Figure S1.** $^1$H NMR spectrum of mesogenic monomer LC44.

4-(but-3-en-1-yloxy)phenyl 4-(but-3-en-1-yloxy)benzoate (LC44): $^1$H NMR(400 MHz, Chloroform-*d*, shown in Figure S1) δ 8.13 (d, *J* = 8.9 Hz, 2H), 7.10 (d, *J* = 9.0 Hz, 2H), 6.94 (dd, *J* = 17.4, 8.9 Hz, 4H), 5.97 – 5.85 (m, 2H), 5.23 – 5.09 (m, 4H), 4.06 (dt, *J* = 32.1, 6.7 Hz, 4H), 2.57 (dq, *J* = 13.5, 6.7 Hz, 4H).

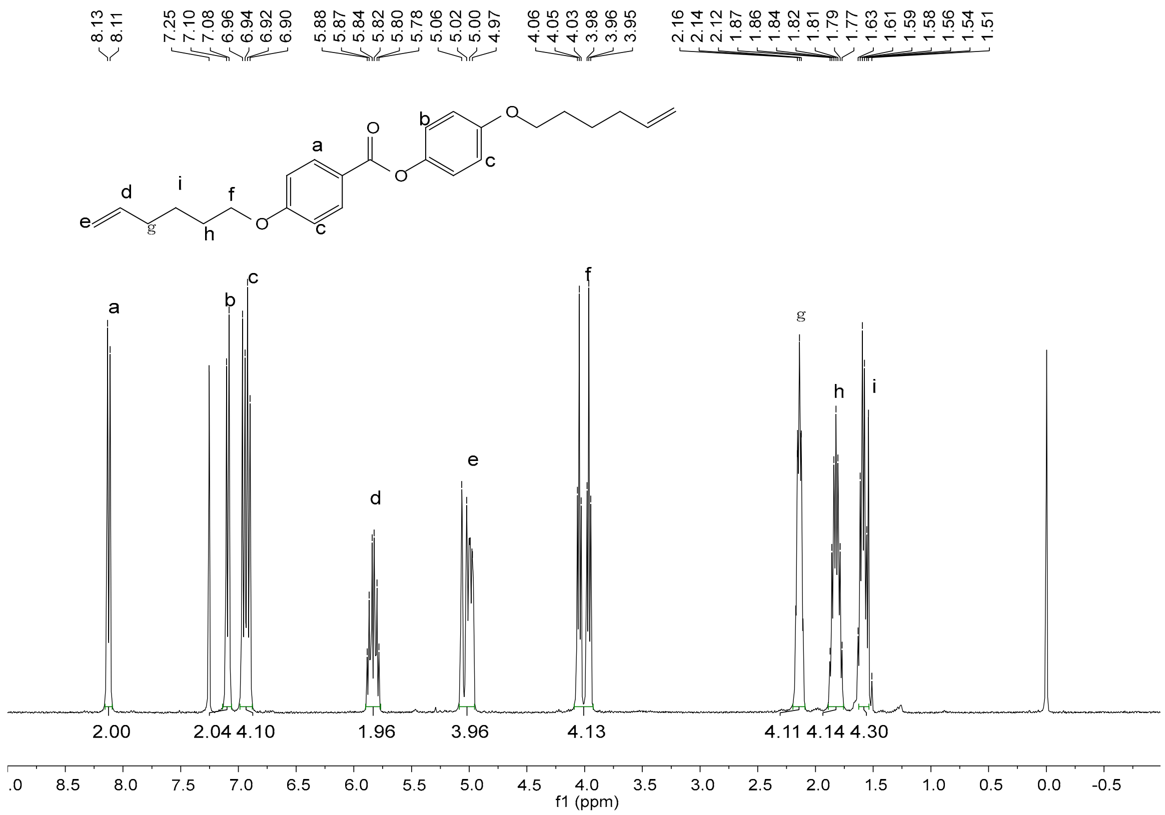


**Figure S2.** $^1$H NMR spectrum of mesogenic monomer LC66.

4-(hex-5-en-1-yloxy)phenyl 4-(hex-5-en-1-yloxy)benzoate (LC66): $^1$H NMR (400 MHz, Chloroform-*d*, shown in Figure S4) δ 8.12 (d, *J* = 8.9 Hz, 2H), 7.09 (d, *J* = 9.0 Hz, 2H), 6.93 (dd, *J* = 17.5, 8.9 Hz, 4H), 5.83 (td, *J* = 16.9, 6.7 Hz, 2H), 5.09 – 4.95 (m, 4H), 4.00 (dt, *J* = 33.2, 6.4 Hz, 4H), 2.13 (d, *J* = 6.4 Hz, 4H), 1.82 (dp, *J* = 14.5, 6.5 Hz, 4H), 1.58 (dt, *J* = 13.9, 7.0 Hz, 4H).

**S-II: Preparation of main-chain polyhydrosiloxane LCEs with 1D programmed shapes.**

The preparation protocol of strip-shaped LCE is illustrated in Scheme S1. The precursor reactant solution was poured into a Teflon rectangular parallel-piped mold with the dimensions of 85mm×12mm×10mm, and then the catalyst solution was added. The mold was executed by ultrasonic process in a laboratory ultrasonic cleaner (KQ-250E, 40 KHz, 250W, Kun Shan Ultrasonic Instruments Co., Ltd.) at 25 °C of ambient temperature for about 30 minutes. A swollen partly crosslinked gel elastomer was generated during this first crosslinking step. By using several milliliters of hexane as remover, the swollen gel was removed from the mold and dried for several hours to let the contained toluene evaporated, and

hence became a partly crosslinked polydomain elastomer. Then the elastomer was slowly uniaxially stretched by a load of 10 g weight for several hours to attain a stable length. Thereafter the elastomer with the stretching load was hung inside a thermostat oven at 60 °C for one day to complete the second step crosslinking to obtain a main-chain LCE strip with rectangular shape.

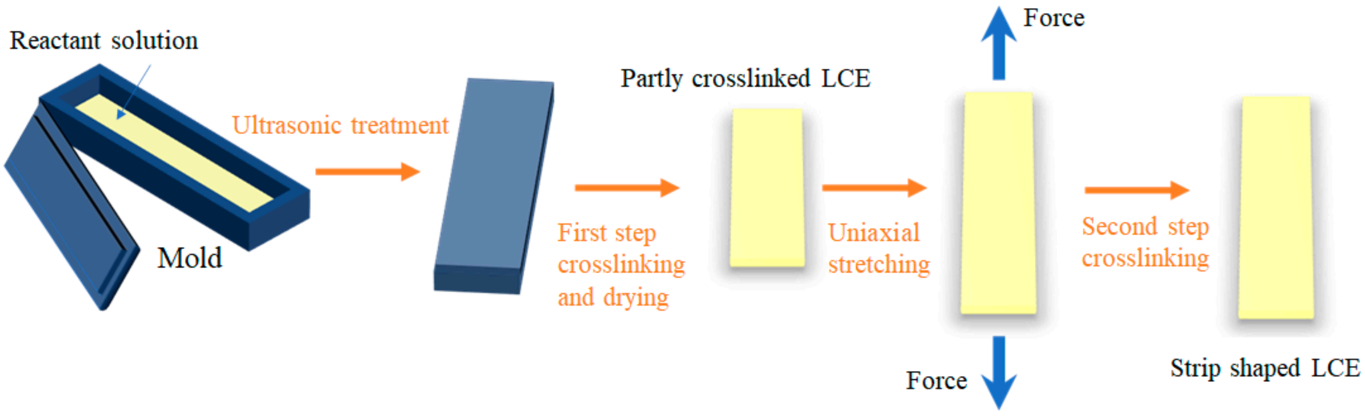


**Scheme S1.** Illustration of the preparation protocol of strip-shaped LCE

The preparation protocol of rod-shaped LCE is illustrated in Scheme S2. The operation of first crosslinking step and drying process were consistent with that in the preparation of strip-shaped LCE, but the used Teflon mold was in cylindrical structure with the diameter and depth of the round hollow being 7mm and 25mm respectively. After the drying process, the partly crosslinked polydomain elastomer was uniaxially compressed by using a counterpoise of 40 g weight for several hours to attain a stable height. Then the elastomer with the compressing load was set inside the thermostat oven at 60 °C for one day to complete the second step crosslinking to obtain a main-chain LCE with a round rod shape.

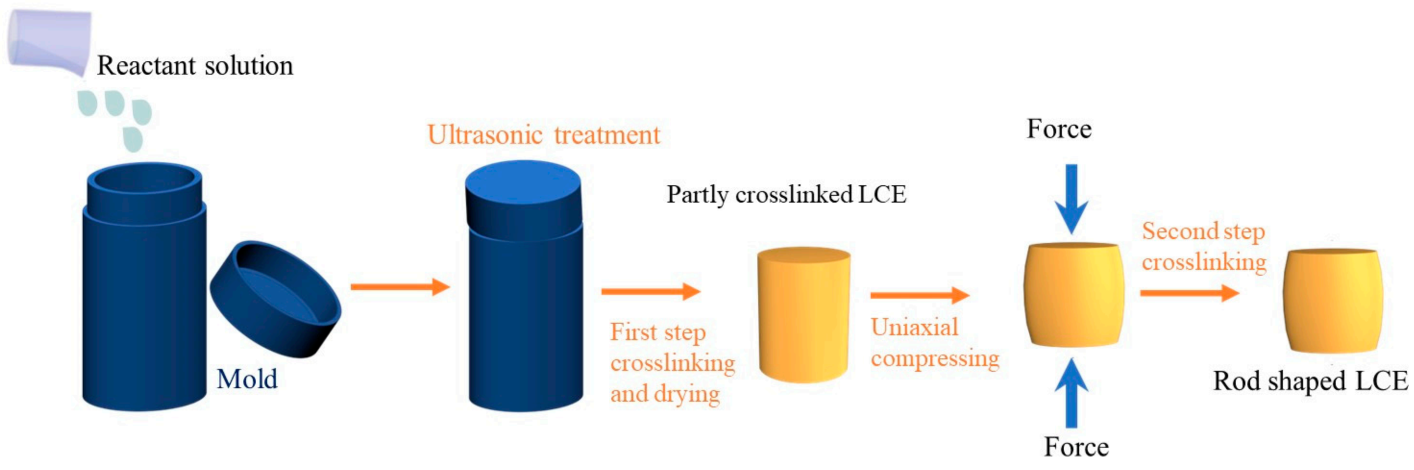


**Scheme S2.** Illustration of the preparation protocol of rod-shaped LCE

**S-III: Preparation of main-chain polyhydrosiloxane LCEs with 2D programmed shapes.**

As illustrated in Scheme S3. The operation of first crosslinking step and drying process were consistent with that in the preparation of strip-shaped LCE, but the used Teflon mold was in cylindrical structure with the diameter and depth of the round hollow being 30mm and 8mm respectively. After the drying process, the partly crosslinked disc-shaped polydomain elastomer was stretched from six, four or three points, evenly-spaced on its circumference along the six, four or three radial directions with mutual angles of 60°, 90°, or 120° under a constant tensile force provided by 10 g of load in each direction. After overnight time of stretching, the disc-shaped elastomer became six-pointed star, four-pointed star or regular triangle shape. The reshaped elastomer with the stretching loads was set inside the thermostat oven at 60 °C for one day to complete the second step crosslinking to obtain the main-chain LCE with a fixed shape of six-pointed star, four-pointed star or regular triangle.

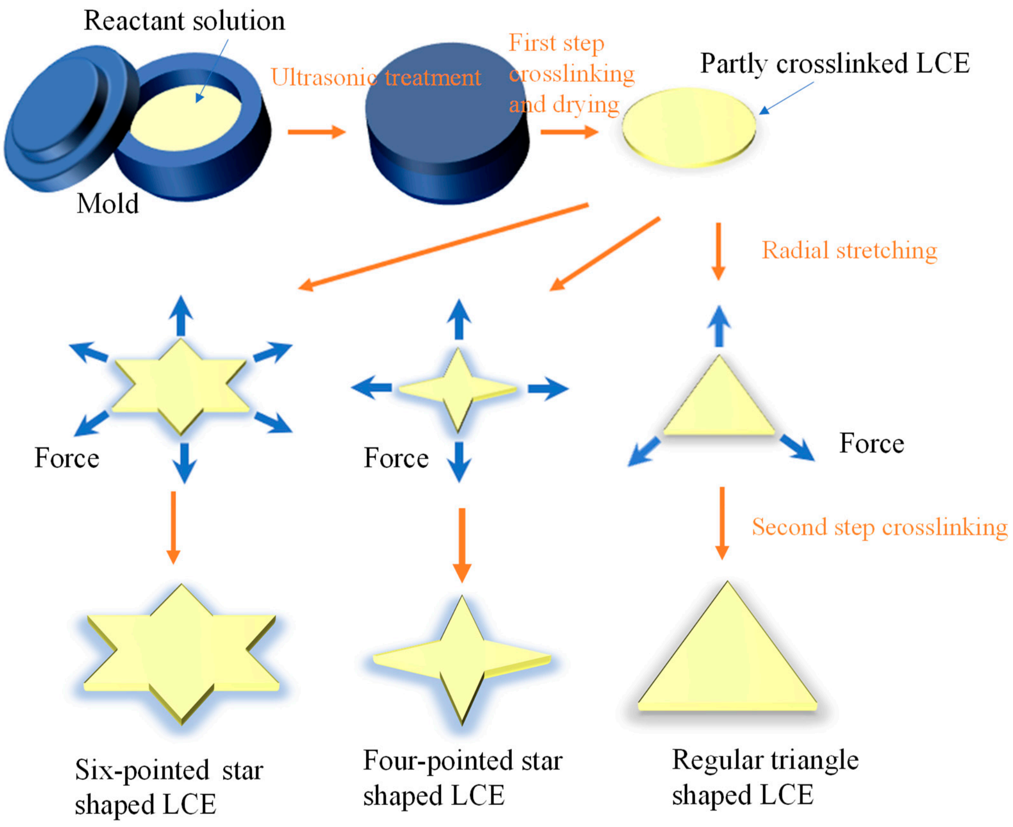


**Scheme S3.** Illustration of the preparation protocol of LCE with a shape of six-pointed star, four-pointed star or regular triangle

**S-IV: Preparation of main-chain polyhydrosiloxane LCEs with 3D programmed shapes.**

The used Teflon mold was the same as above in the preparation of LCEs with 2D programmed shapes.

The preparation protocol of “3D-S-LCE” is illustrated in Scheme S4. A disc tablet badge with an 3D pattern of Heilongjiang university logo was pre-set on the bottom of the mold. The operation of first crosslinking step and drying process were consistent with that in the preparation of LCEs with 2D programmed shapes. The obtained partly crosslinked polydomain elastomer was disc-shape with a stamped 3D pattern, imprinted by the tablet badge, on its lower surface. The stamping patterned surface of the partly crosslinked elastomer was pressed by a counterpoise of 50 g weight through a flat Teflon plate for several hours to become flat. Then the elastomer with the pressing load was set inside the thermostat oven at 60 °C

for one day to complete the second step crosslinking and fix the flattened surface, and hence the "3D-S-LCE" was obtained.

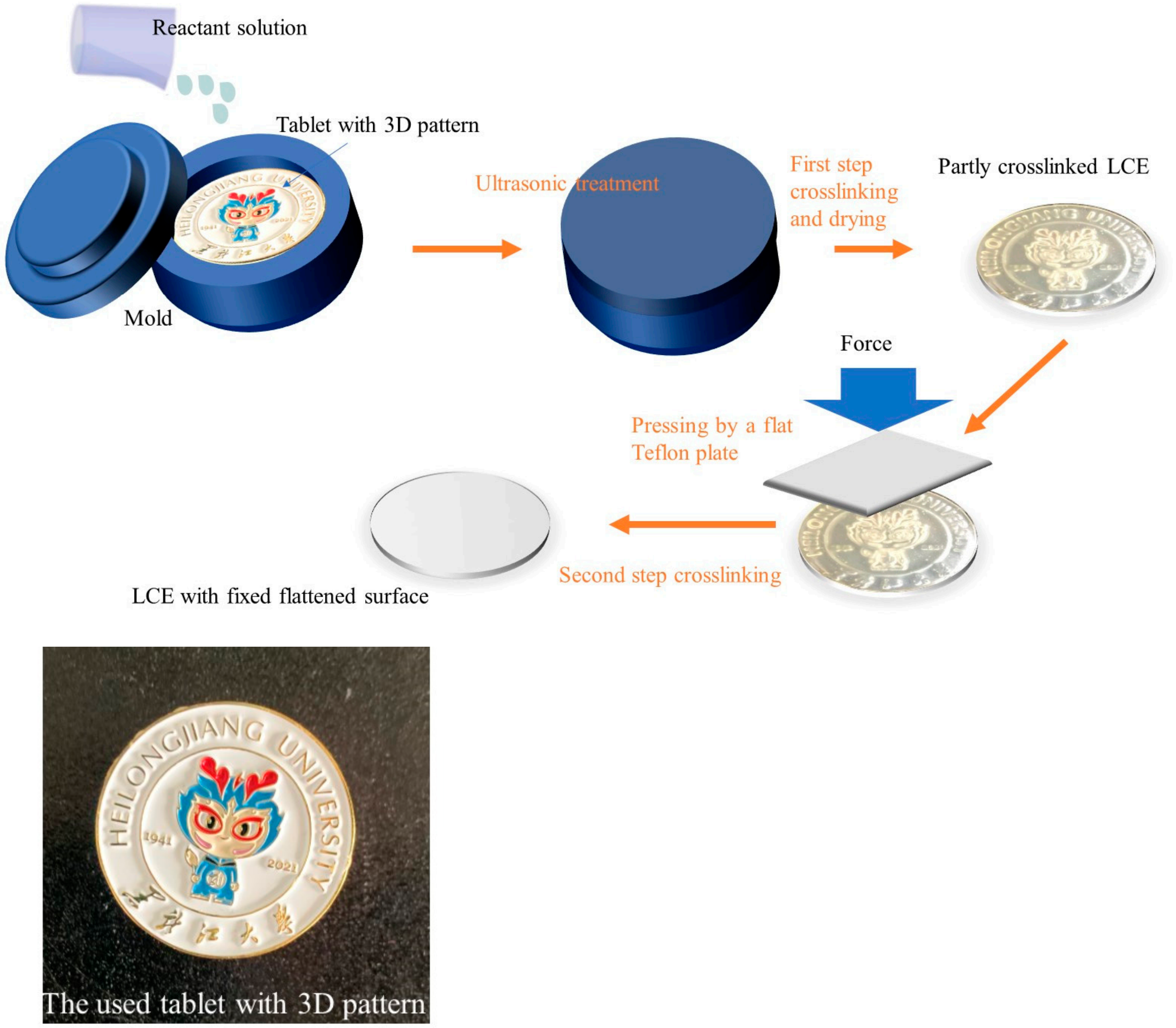


**Scheme S4.** Illustration of the preparation protocol of 3D-S-LCE

The preparation protocol of "3D-E-LCE" is illustrated in Scheme S5. The operation of first crosslinking step and drying process were consistent with that in the preparation of LCEs with 2D programmed shapes. After the drying process, the upper surface of the partly crosslinked disc-shaped polydomain elastomer was pressed by a 3D plum blossom plastic mould (No.1 plastic mould), which carried a counterpoise of 50 g weight. While the lower surface of the partly crosslinked disc-shaped elastomer was

carried by a layer of plasticene for stress buffering. After about 10 hours of pressing, a 3D plum blossom was embossed on the elastomer upper surface. Then the elastomer with the embosser pressing load was set inside the thermostat oven at 60 °C for one day to complete the second step crosslinking and fix the 3D plum blossom pattern, and hence the "3D-E-LCE" was obtained.

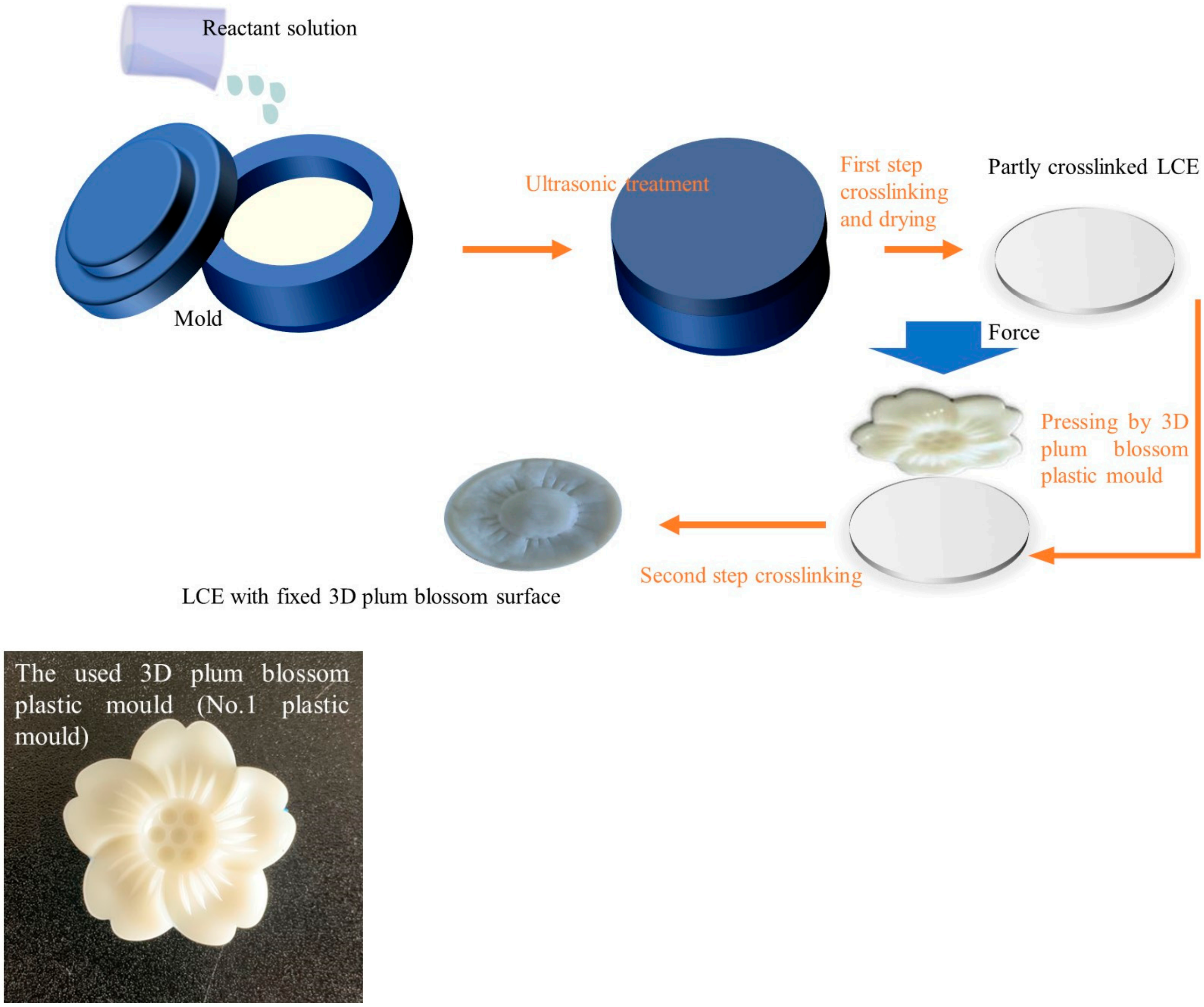


**Scheme S5.** Illustration of the preparation protocol of 3D-E-LCE

The preparation protocol of "3D-SE-LCE" is illustrated in Scheme S6. The operation of first crosslinking step and drying process were consistent with that in the preparation of "3D-S-LCE". After the drying process, the surface of the partly crosslinked disc-shaped polydomain elastomer, with a stamped 3D pattern of Heilongjiang university logo, was pressed by another 3D plum blossom plastic mould (No.2

plastic mould) as above operation in preparation of "3D-E-LCE". Lastly, the initial stamped 3D pattern on elastomer surface was replaced by the embossed 3D plum blossom pattern from No.2 plastic mould. Then the elastomer with the embosser pressing load was set inside the thermostat oven at 60 °C for one day to complete the second step crosslinking and fix the 3D plum blossom pattern, and hence the "3D-SE-LCE" was obtained.

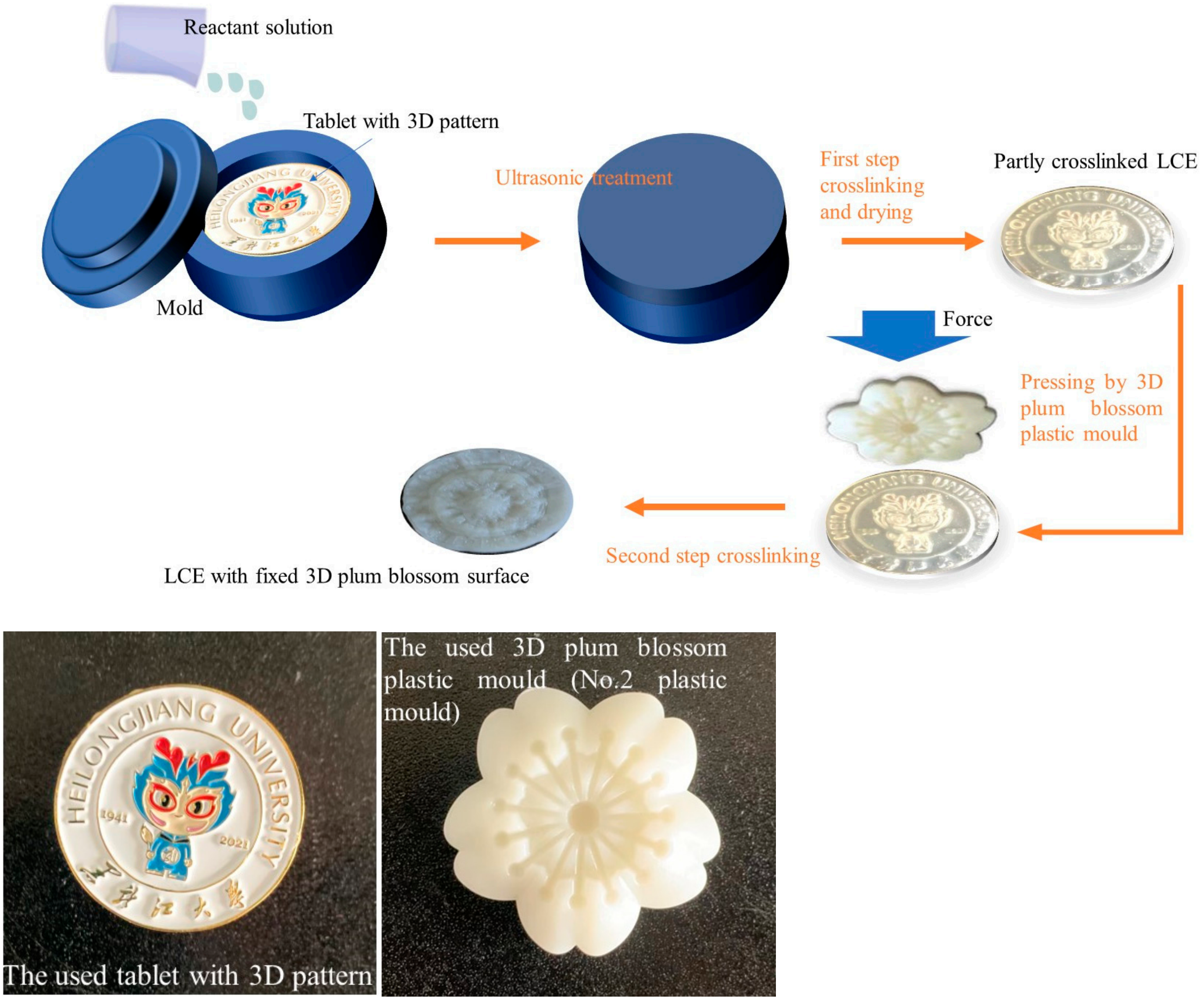


**Scheme S6.** Illustration of the preparation protocol of 3D-SE-LCE

**S-V: The calculation of actuation fixity of the LCEs with 1D shape changing performances.**

The calculation of actuation fixity of the strip-shaped LCE can be illustrated as in Scheme S7. As described in "S-I", after the first step crosslinking and drying process, a partly crosslinked polydomain LCE

with a rectangle strip shape was generated. The length of the sample at this stage was denoted as the initial length $L_0$. The sample was then uniaxially stretched to a desired length $L_1$. After the second step crosslinking and removement of the stretching load, a rectangle strip-shaped LCE was obtained. The length of the sample at this stage was denoted as the cured length $L_2$. The sample was heated to above the nematic-isotropic temperature ($T_{ni}$), and hence axially contracted to be a length "$L_3$". Then the sample was cooled to be in room temperature, and hence axially elongated to be a length "$L_4$".

The cure fixity is calculated as: $f_{cure} = L_2 / L_1$. The heating deformation fixity is calculated as: $f_{heat} = L_0 / L_3$. The cooling deformation fixity is calculated as: $f_{cool} = L_4 / L_2$.

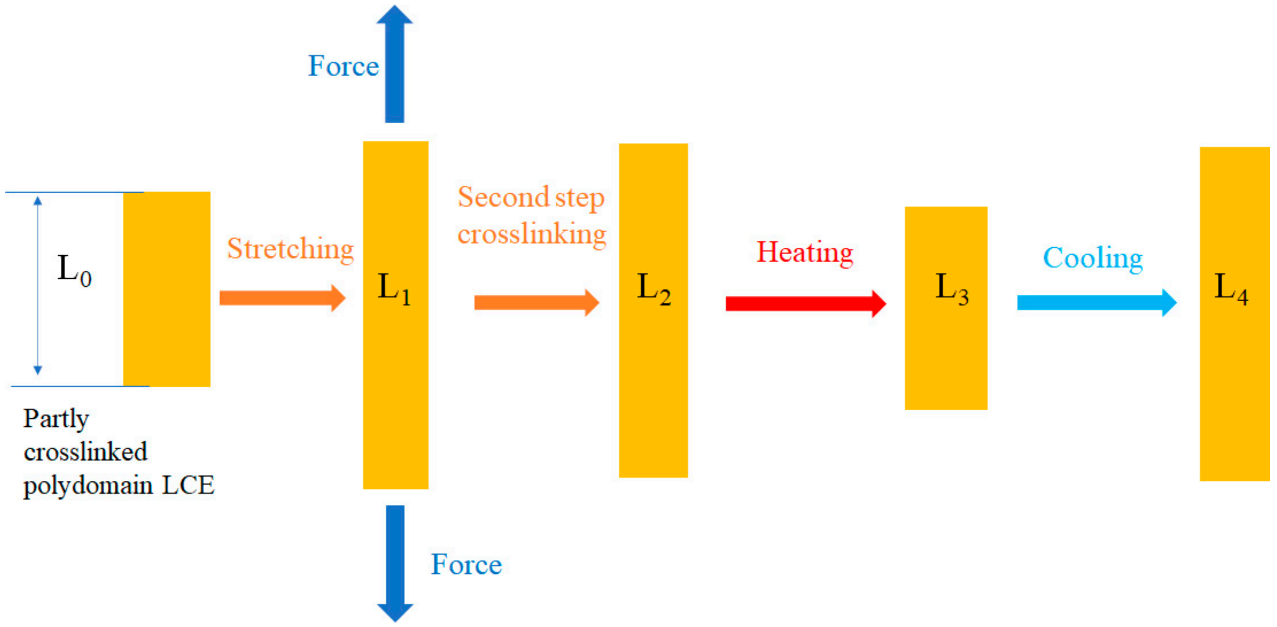


**Scheme S7.** Illustration for the calculation of actuation fixity of the strip-shaped LCE

The calculation of actuation fixity of the rod-shaped LCE can be illustrated as in Scheme S8. As described in "S-I", after the first step crosslinking and drying process, a partly crosslinked polydomain LCE with a round rod shape was generated. The height of the sample at this stage was denoted as the initial height $h_0$. The sample was then uniaxially compressed to be a desired height $h_1$. After the second step crosslinking and removement of the compressing load, a shortened rod-shaped LCE was obtained. The height of the sample at this stage was denoted as the cured height $h_2$. The sample was heated to above the nematic-isotropic temperature ($T_{ni}$), and hence axially heightened to be a height "$h_3$". Then the sample was

cooled to be in room temperature, and hence axially shortened to be a height "$h_4$".

The cure fixity is calculated as: $f_{cure} = h_1 / h_2$. The heating deformation fixity is calculated as: $f_{heat} = h_3 / h_0$. The cooling deformation fixity is calculated as: $f_{cool} = h_2 / h_4$.

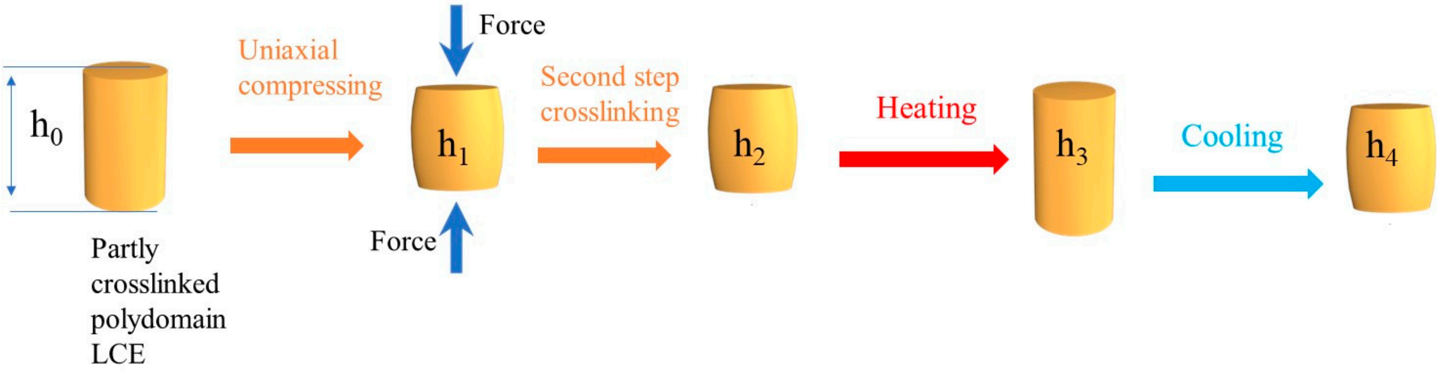


**Scheme S8.** Illustration for the calculation of actuation fixity of the rod-shaped LCE
